# Queen Mary's Convalescent Auxiliary Hospitals

**Published:** 1915-05-29

**Authors:** Kathleen Falmouth, M. E. Gwynne Holford

**Affiliations:** 2 St. James's Square, S.W.; 22 Wilton Street, S.W.


### THE EDITOR'S LETTER-BOX.

### Queen Mary's Convalescent Auxiliary Hospitals.

*To the Editor of* The Hospital.

Sir,—Having recently, through the courtesy of the Press, been able to make known the urgent need of convalescent hospitals for those who have lost their limbs in the war, we beg to inform your readers that Roehampton House (near London) has been acquired and will shortly be opened for this purpose, and that Mr. J. Pierpont Morgan has generously offered Dover House (almost adjoining) for the use of officers. These houses together are capable of accommodating about 300 cases. Her Majesty Queen Mary has graciously consented to the hospitals being named "Queen Mary's Convalescent Auxiliary Hospitals," and has given a donation of £200.

At these convalescent hospitals our brave men will be cared for until they have recovered their strength and nerve; and having learned to use their artificial limbs, they will again be capable of taking up employment in the form best suited to each individual. Working in conjunction with other societies, every effort will be made to fit the men to earn their own living in the future. To enable this urgent work to proceed without delay, grants have been made by the National Relief Fund and the Red Cross Society. But we need a large sum in addition for the equipment, rent, and maintenance of the hospitals. It is for these gallant men—sons of our Empire—that we earnestly appeal for funds to carry out the work efficiently. £50 will maintain for a year a bed to be named after the donor, and it is hoped that donations of this amount will be forthcoming from many quarters—including industrial firms—to secure the provision of county beds, beds for naval, military, and aircraft units, and also for men from our overseas Dominions. Communications and donations should be addressed to C. H. Kenderdine, Esq. (marked "Auxiliary Hospital"), at St. Stephen's House, Westminster, who will be pleased to answer all inquiries.—Yours obediently,

Kathleen Falmouth,
2 St. James's Square, S.W.
M. E. Gwynne Holford,
22 Wilton Street, S.W.

May 19, 1915.

### The Annual Dinner and the War.

*To the Editor of* The Hospital.

